# Supplementary material for: Whole-genome analysis of extensively drug-resistant Acinetobacter baumannii isolates from a Peruvian tertiary hospital reveals the emergence of OXA-23-producing ST79 and ST1079 clones
Source: Microbiol Spectr. 2026 Feb 3;14(3):e03415-25. doi: 10.1128/spectrum.03415-25 (PMC12955417; doi:10.1128/spectrum.03415-25)
Supplement: Table S3 — Virulence mechanism groups and associated genes identified in Acinetobacter baumannii isolates. [file spectrum.03415-25-s0003.docx]

| **Table S3.** Virulence mechanism groups and associated genes identified in *Acinetobacter baumannii* isolates | | |
| --- | --- | --- |
| **Isolate ID** | **Virulence mechanism group** | **Virulence genes** |
| H2M2401 | Adherence | *ata, fimT, fimU, fimV, gspO/pilD, pilB, pilC, pilF, pilG, pilH, pilI, pilJ, pilM, pilN, pilO, pilP, pilQ, pilR, pilS, pilT, pilU, pilV, pilW, pilX, pilY1, tsaP* |
|  | Quorum sensing and biofilm | *abaI, abaR, adeF, adeG, adeH, bap, csuA, csuA/B, csuB, csuC, csuD, csuE, pgaA, pgaB, pgaC, pgaD* |
|  | Secretion system | *clpV/tssH, gspC, gspD, gspE1, gspE2, gspF, gspG, gspH, gspI, gspK, gspL, gspM, gspN, hcp/tssD, tagX, tssA, tssB, tssC, tssE, tssF, tssG, tssK, tssL, tssM* |
|  | Exotoxin | *plc1, plcD, plc2* |
|  | Immune modulation | *galE, lpsB, lpxA, lpxB, lpxC, lpxD, lpxL, lpxM, pbpG* |
|  | Iron uptake systems | *barA, barB, basA, basB, basC, basD, basF, basG, basH, basI, basJ, bauB, bauC, bauD, bauE, bauF, entE, hemO* |
|  | Two-component systems | *bfmR, bfmS* |
| H2M2402 | Adherence | *ata, fimT, fimU, fimV, gspO/pilD, pilB, pilC, pilF, pilG, pilH, pilI, pilJ, pilM, pilN, pilO, pilP, pilQ, pilR, pilS, pilT, pilU, pilV, pilW, pilX, pilY1, tsaP* |
|  | Quorum sensing and biofilm | *abaI, abaR, adeF, adeG, adeH, bap, csuA, csuA/B, csuB, csuC, csuD, csuE, pgaA, pgaB, pgaC, pgaD* |
|  | Secretion system | *clpV/tssH, gspC, gspD, gspE1, gspE2, gspF, gspG, gspH, gspI, gspK, gspL, gspM, gspN, hcp/tssD, tagX, tssA, tssB, tssC, tssE, tssF, tssG, tssK, tssL, tssM* |
|  | Exotoxin | *plc1, plc2, plcD* |
|  | Immune modulation | *galE, lpsB, lpxA, lpxB, lpxC, lpxD, lpxL, lpxM, pbpG* |
|  | Iron uptake systems | *barA, barB, basA, basB, basC, basD, basF, basG, basH, basI, basJ, bauB, bauC, bauD, bauE, bauF, entE, hemO* |
|  | Two-component systems | *bfmR, bfmS* |
| H2M2403 | Adherence | *fimT, fimU, fimV, gspO/pilD, pilA, pilB, pilC, pilF, pilG, pilH, pilI, pilJ, pilM, pilN, pilO, pilP, pilQ, pilR, pilS, pilT, pilU, pilV, pilW, pilX, pilY1, tsaP* |
|  | Quorum sensing and biofilm | *abaI, adeF, adeG, adeH, csuA, csuA/B, csuB, csuC, csuD, csuE, pgaA, pgaB, pgaC, pgaD* |
|  | Secretion system | *clpV/tssH, gspC, gspE1, gspE2, gspF, gspG, gspH, gspI, gspK, gspL, gspM, gspN, hcp/tssD, tagX, tssA, tssB, tssC, tssE, tssF, tssG, tssK, tssL, tssM* |
|  | Exotoxin | *plc1, plc2, plcD* |
|  | Immune modulation | *galE, lpsB, lpxA, lpxB, lpxC, lpxD, lpxL, lpxM, ompA, pbpG, pgi* |
|  | Iron uptake systems | *barA, barB, basA, basB, basC, basD, basF, basG, basH, basI, basJ, bauA, bauB, bauC, bauD, bauE, bauF, entE* |
|  | Two-component systems | *bfmR, bfmS* |
| H2M2404 | Adherence | *fimT, fimU, fimV, gspO/pilD, pilA, pilB, pilC, pilE, pilF, pilG, pilH, pilI, pilJ, pilM, pilN, pilO, pilP, pilQ, pilR, pilS, pilT, pilU, pilV, pilW, pilX, pilY1, tsaP* |
|  | Quorum sensing and biofilm | *abaI, abaR, adeF, adeG, adeH, csuA, csuA/B, csuB, csuC, csuD, csuE, pgaA, pgaB, pgaC, pgaD* |
|  | Secretion system | *clpV/tssH, gspC, gspD, gspE1, gspE2, gspF, gspG, gspH, gspI, gspK, gspL, gspM, gspN, hcp/tssD, tagX, tssA, tssB, tssC, tssE, tssF, tssG, tssK, tssL, tssM, vgrG/tssI* |
|  | Exotoxin | *plc1, plc2, plcD* |
|  | Immune modulation | *galE, lpsB, lpxA, lpxB, lpxC, lpxD, lpxL, lpxM, ompA, pbpG* |
|  | Iron uptake systems | *barA, barB, basA, basB, basC, basD, basF, basG, basH, basI, basJ, bauA, bauB, bauC, bauD, bauE, bauF, entE* |
|  | Two-component systems | *bfmR, bfmS* |
| H2M2405 | Adherence | *ata, fimT, fimU, fimV, gspO/pilD, pilB, pilC, pilF, pilG, pilH, pilI, pilJ, pilM, pilN, pilO, pilP, pilQ, pilR, pilS, pilT, pilU, pilV, pilW, pilX, pilY1, tsaP* |
|  | Quorum sensing and biofilm | *abaI, abaR, adeF, adeG, adeH, bap, csuA, csuA/B, csuB, csuC, csuD, csuE, pgaA, pgaB, pgaC, pgaD* |
|  | Secretion system | *clpV/tssH, gspC, gspD, gspE1, gspE2, gspF, gspG, gspH, gspI, gspK, gspL, gspM, gspN, hcp/tssD, tagX, tssA, tssB, tssC, tssE, tssF, tssG, tssK, tssL, tssM* |
|  | Exotoxin | *plc1, plc2, plcD* |
|  | Immune modulation | *galE, lpsB, lpxA, lpxB, lpxC, lpxD, lpxL, lpxM, pbpG* |
|  | Iron uptake systems | *barA, barB, basA, basB, basC, basD, basF, basG, basH, basI, basJ, bauB, bauC, bauD, bauE, bauF, entE, hemO* |
|  | Two-component systems | *bfmR, bfmS* |
| H2M2406 | Adherence | *fimT, fimU, fimV, gspO/pilD, pilA, pilB, pilC, pilE, pilF, pilG, pilH, pilI, pilJ, pilM, pilN, pilO, pilP, pilQ, pilR, pilS, pilT, pilU, pilV, pilW, pilX, pilY1, tsaP* |
|  | Quorum sensing and biofilm | *abaI, abaR, adeF, adeG, adeH, bap, csuA, csuA/B, csuB, csuC, csuD, csuE, pgaA, pgaB, pgaC, pgaD* |
|  | Secretion system | *clpV/tssH, gspC, gspD, gspE1, gspE2, gspF, gspG, gspH, gspI, gspK, gspL, gspM, gspN, hcp/tssD, tagX, tssA, tssB, tssC, tssE, tssF, tssG, tssK, tssL, tssM, vgrG/tssI* |
|  | Exotoxin | *plc1, plc2, plcD* |
|  | Immune modulation | *galE, lpsB, lpxA, lpxB, lpxC, lpxD, lpxL, lpxM, ompA, pbpG* |
|  | Iron uptake systems | *barA, barB, basA, basB, basC, basD, basF, basG, basH, basI, basJ, bauA, bauB, bauC, bauD, bauE, bauF, entE* |
|  | Two-component systems | *bfmR, bfmS* |
| H2M2407 | Adherence | *fimT, fimU, fimV, gspO/pilD, pilA, pilB, pilC, pilE, pilF, pilG, pilH, pilI, pilJ, pilM, pilN, pilO, pilP, pilQ, pilR, pilS, pilT, pilU, pilV, pilW, pilX, pilY1, tsaP* |
|  | Quorum sensing and biofilm | *abaI, abaR, adeF, adeG, adeH, bap, csuA, csuA/B, csuB, csuC, csuD, csuE, pgaA, pgaB, pgaC, pgaD* |
|  | Secretion system | *clpV/tssH, gspC, gspD, gspE1, gspE2, gspF, gspG, gspH, gspI, gspK, gspL, gspM, gspN, hcp/tssD, tagX, tssA, tssB, tssC, tssE, tssF, tssG, tssK, tssL, tssM, vgrG/tssI)* |
|  | Exotoxin | *plc1, plc2, plcD* |
|  | Immune modulation | *galE, galU, lpsB, lpxA, lpxB, lpxC, lpxD, lpxL, lpxM, ompA, pbpG, pgi, pseB, pseC, pseF, pseG, pseH, pseI, tviB* |
|  | Iron uptake systems | *barA, barB, basA, basB, basC, basD, basF, basG, basH, basI, basJ, bauA, bauB, bauC, bauD, bauE, bauF, entE* |
|  | Two-component systems | *bfmR, bfmS* |
| H2M2408 | Adherence | *fimT, fimU, fimV, gspO/pilD, pilA, pilB, pilC, pilE, pilF, pilG, pilH, pilI, pilJ, pilM, pilN, pilO, pilP, pilQ, pilR, pilS, pilT, pilU, pilV, pilW, pilX, pilY1, tsaP* |
|  | Quorum sensing and biofilm | *abaI, abaR, adeF, adeG, adeH, bap, csuA, csuA/B, csuB, csuC, csuD, csuE, pgaA, pgaB, pgaC, pgaD* |
|  | Secretion system | *clpV/tssH, gspC, gspD, gspE1, gspE2, gspF, gspG, gspH, gspI, gspK, gspL, gspM, gspN, hcp/tssD, tagX, tssA, tssB, tssC, tssE, tssF, tssG, tssK, tssL, tssM, vgrG/tssI* |
|  | Exotoxin | *plc1, plc2, plcD* |
|  | Immune modulation | *galE, galU, lpsB, lpxA, lpxB, lpxC, lpxD, lpxL, lpxM, ompA, pbpG, pgi, pseB, pseC, pseF, pseG, pseH, pseI, tviB* |
|  | Iron uptake systems | *barA, barB, basA, basB, basC, basD, basF, basG, basH, basI, basJ, bauA, bauB, bauC, bauD, bauE, bauF, entE* |
|  | Two-component systems | *bfmR, bfmS* |
| H2M2501 | Adherence | *fimT, fimU, fimV, gspO/pilD, pilA, pilB, pilC, pilE, pilF, pilG, pilH, pilI, pilJ, pilM, pilN, pilO, pilP, pilQ, pilR, pilS, pilT, pilU, pilV, pilW, pilX, pilY1, tsaP* |
|  | Quorum sensing and biofilm | *abaI, abaR, adeF, adeG, adeH, csuA, csuA/B, csuB, csuC, csuD, csuE, pgaA, pgaB, pgaC, pgaD* |
|  | Secretion system | *clpV/tssH, gspC, gspD, gspE1, gspE2, gspF, gspG, gspH, gspI, gspK, gspL, gspM, gspN, hcp/tssD, tagX, tssA, tssB, tssC, tssE, tssF, tssG, tssK, tssL, tssM, vgrG/tssI* |
|  | Exotoxin | *plc1, plc2, plcD* |
|  | Immune modulation | *galE, galU, lpsB, lpxA, lpxB, lpxC, lpxD, lpxL, lpxM, ompA, pbpG, pgi, pseB, pseC, pseF, pseG, pseH, pseI, tviB* |
|  | Iron uptake systems | *barA, barB, basA, basB, basC, basD, basF, basG, basH, basI, basJ, bauA, bauB, bauC, bauD, bauE, bauF, entE* |
|  | Two-component systems | *bfmR, bfmS* |
| H2M2502 | Adherence | *fimT, fimU, fimV, gspO/pilD, pilA, pilB, pilC, pilE, pilF, pilG, pilH, pilI, pilJ, pilM, pilN, pilO, pilP, pilQ, pilR, pilS, pilT, pilU, pilV, pilW, pilX, pilY1, tsaP* |
|  | Quorum sensing and biofilm | *abaI, abaR, adeF, adeG, adeH, csuA, csuA/B, csuB, csuC, csuD, csuE, pgaA, pgaB, pgaC, pgaD* |
|  | Secretion system | *clpV/tssH, gspC, gspD, gspE1, gspE2, gspF, gspG, gspH, gspI, gspK, gspL, gspM, gspN, hcp/tssD, tagX, tssA, tssB, tssC, tssE, tssF, tssG, tssK, tssL, tssM, vgrG/tssI* |
|  | Exotoxin | *plc1, plc2, plcD* |
|  | Immune modulation | *galE, lpsB, lpxA, lpxB, lpxC, lpxD, lpxL, lpxM, ompA, pbpG* |
|  | Iron uptake systems | *barA, barB, basA, basB, basC, basD, basF, basG, basH, basI, basJ, bauA, bauB, bauC, bauD, bauE, bauF, entE* |
|  | Two-component systems | *bfmR, bfmS* |
| H2M2503 | Adherence | *fimT, fimU, fimV, gspO/pilD, pilA, pilB, pilC, pilE, pilF, pilG, pilH, pilI, pilJ, pilM, pilN, pilO, pilP, pilQ, pilR, pilS, pilT, pilU, pilV, pilW, pilX, pilY1, tsaP* |
|  | Quorum sensing and biofilm | *abaI, abaR, adeF, adeG, adeH, csuA, csuA/B, csuB, csuC, csuD, csuE, pgaA, pgaB, pgaC, pgaD* |
|  | Secretion system | *clpV/tssH, gspC, gspD, gspE1, gspE2, gspF, gspG, gspH, gspI, gspK, gspL, gspM, gspN, hcp/tssD, tagX, tssA, tssB, tssC, tssE, tssF, tssG, tssK, tssL, tssM, vgrG/tssI* |
|  | Exotoxin | *plc1, plc2, plcD* |
|  | Immune modulation | *galE, galU, lpsB, lpxA, lpxB, lpxC, lpxD, lpxL, lpxM, ompA, pbpG, pgi, pseB, pseC, pseF, pseG, pseH, pseI, tviB* |
|  | Iron uptake systems | *barA, barB, basA, basB, basC, basD, basF, basG, basH, basI, basJ, bauA, bauB, bauC, bauD, bauE, bauF, entE* |
|  | Two-component systems | *bfmR, bfmS* |
| H2M2504 | Adherence | *fimT, fimU, fimV, gspO/pilD, pilA, pilB, pilC, pilF, pilG, pilH, pilI, pilJ, pilM, pilN, pilO, pilP, pilQ, pilR, pilS, pilT, pilU, pilV, pilW, pilX, pilY1, tsaP* |
|  | Quorum sensing and biofilm | *abaI, adeF, adeG, adeH, csuA, csuA/B, csuB, csuC, csuD, csuE, pgaA, pgaB, pgaC, pgaD* |
|  | Secretion system | *clpV/tssH, gspC, gspE1, gspE2, gspF, gspG, gspH, gspI, gspK, gspL, gspM, gspN, hcp/tssD, tagX, tssA, tssB, tssC, tssE, tssF, tssG, tssK, tssL, tssM* |
|  | Exotoxin | *plc1, plc2, plcD* |
|  | Immune modulation | *galE, lpsB, lpxA, lpxB, lpxC, lpxD, lpxL, lpxM, ompA, pbpG, pgi* |
|  | Iron uptake systems | *barA, barB, basA, basB, basC, basD, basF, basG, basH, basI, basJ, bauA, bauB, bauC, bauD, bauE, bauF, entE* |
|  | Two-component systems | *bfmR, bfmS* |
| H2M2505 | Adherence | *fimT, fimU, fimV, gspO/pilD, pilA, pilB, pilC, pilE, pilF, pilG, pilH, pilI, pilJ, pilM, pilN, pilO, pilP, pilQ, pilR, pilS, pilT, pilU, pilV, pilW, pilX, pilY1, tsaP* |
|  | Quorum sensing and biofilm | *abaI, abaR, adeF, adeG, adeH, csuA, csuA/B, csuB, csuC, csuD, csuE, pgaA, pgaB, pgaC, pgaD* |
|  | Secretion system | *clpV/tssH, gspC, gspD, gspE1, gspE2, gspF, gspG, gspH, gspI, gspK, gspL, gspM, gspN, hcp/tssD, tagX, tssA, tssB, tssC, tssE, tssF, tssG, tssK, tssL, tssM, vgrG/tssI* |
|  | Exotoxin | *plc1, plc2, plcD* |
|  | Immune modulation | *galE, galU, lpsB, lpxA, lpxB, lpxC, lpxD, lpxL, lpxM, ompA, pbpG, pgi, pseB, pseC, pseF, pseG, pseH, pseI, tviB* |
|  | Iron uptake systems | *barA, barB, basA, basB, basC, basD, basF, basG, basH, basI, basJ, bauA, bauB, bauC, bauD, bauE, bauF, entE* |
|  | Two-component systems | *bfmR, bfmS* |
| H2M2506 | Adherence | *fimT, fimU, fimV, gspO/pilD, pilA, pilB, pilC, pilE, pilF, pilG, pilH, pilI, pilJ, pilM, pilN, pilO, pilP, pilQ, pilR, pilS, pilT, pilU, pilV, pilW, pilX, pilY1, tsaP* |
|  | Quorum sensing and biofilm | *abaI, abaR, adeF, adeG, adeH, csuA, csuA/B, csuB, csuC, csuD, csuE, pgaA, pgaB, pgaC, pgaD* |
|  | Secretion system | *clpV/tssH, gspC, gspD, gspE1, gspE2, gspF, gspG, gspH, gspI, gspK, gspL, gspM, gspN, hcp/tssD, tagX, tssA, tssB, tssC, tssE, tssF, tssG, tssK, tssL, tssM, vgrG/tssI* |
|  | Exotoxin | *plc1, plc2, plcD* |
|  | Immune modulation | *galE, lpsB, lpxA, lpxB, lpxC, lpxD, lpxL, lpxM, ompA, pbpG* |
|  | Iron uptake systems | *barA, barB, basA, basB, basC, basD, basF, basG, basH, basI, basJ, bauA, bauB, bauC, bauD, bauE, bauF, entE* |
|  | Two-component systems | *bfmR, bfmS* |
| H2M2302 | Adherence | *fimT, fimU, fimV, gspO/pilD, pilA, pilB, pilC, pilE, pilF, pilG, pilH, pilI, pilJ, pilM, pilN, pilO, pilP, pilQ, pilR, pilS, pilT, pilU, pilV, pilW, pilX, pilY1, tsaP* |
|  | Quorum sensing and biofilm | *abaI, abaR, adeF, adeG, adeH, csuA, csuA/B, csuB, csuC, csuD, csuE, pgaA, pgaB, pgaC, pgaD* |
|  | Secretion system | *clpV/tssH, gspC, gspD, gspE1, gspE2, gspF, gspG, gspH, gspI, gspK, gspL, gspM, gspN, hcp/tssD, tagX, tssA, tssB, tssC, tssE, tssF, tssG, tssK, tssL, tssM, vgrG/tssI* |
|  | Exotoxin | *plc1, plc2, plcD* |
|  | Immune modulation | *galE, galU, lpsB, lpxA, lpxB, lpxC, lpxD, lpxL, lpxM, ompA, pbpG, pgi, pseB, pseC, pseF, pseG, pseH, pseI, tviB* |
|  | Iron uptake systems | *barA, barB, basA, basB, basC, basD, basF, basG, basH, basI, basJ, bauA, bauB, bauC, bauD, bauE, bauF, entE* |
|  | Two-component systems | *bfmR, bfmS* |
| H2M2303 | Adherence | *fimT, fimU, fimV, gspO/pilD, pilA, pilB, pilC, pilE, pilF, pilG, pilH, pilI, pilJ, pilM, pilN, pilO, pilP, pilQ, pilR, pilS, pilT, pilU, pilV, pilW, pilX, pilY1, tsaP* |
|  | Quorum sensing and biofilm | *abaI, abaR, adeF, adeG, adeH, csuA, csuA/B, csuB, csuC, csuD, csuE, pgaA, pgaB, pgaC, pgaD* |
|  | Secretion system | *clpV/tssH, gspC, gspD, gspE1, gspE2, gspF, gspG, gspH, gspI, gspK, gspL, gspM, gspN, hcp/tssD, tagX, tssA, tssB, tssC, tssE, tssF, tssG, tssK, tssL, tssM, vgrG/tssI* |
|  | Exotoxin | *plc1, plc2, plcD* |
|  | Immune modulation | *galE, lpsB, lpxA, lpxB, lpxC, lpxD, lpxL, lpxM, ompA, pbpG* |
|  | Iron uptake systems | *barA, barB, basA, basB, basC, basD, basF, basG, basH, basI, basJ, bauA, bauB, bauC, bauD, bauE, bauF, entE* |
|  | Two-component systems | *bfmR, bfmS* |
| H2M2304 | Adherence | *fimT, fimU, fimV, gspO/pilD, pilA, pilB, pilC, pilE, pilF, pilG, pilH, pilI, pilJ, pilM, pilN, pilO, pilP, pilQ, pilR, pilS, pilT, pilU, pilV, pilW, pilX, pilY1, tsaP* |
|  | Quorum sensing and biofilm | *abaI, abaR, adeF, adeG, adeH, csuA, csuA/B, csuB, csuC, csuD, csuE, pgaA, pgaB, pgaC, pgaD* |
|  | Secretion system | *clpV/tssH, gspC, gspD, gspE1, gspE2, gspF, gspG, gspH, gspI, gspK, gspL, gspM, gspN, hcp/tssD, tagX, tssA, tssB, tssC, tssE, tssF, tssG, tssK, tssL, tssM, vgrG/tssI* |
|  | Exotoxin | *plc1, plc2, plcD* |
|  | Immune modulation | *galE, lpsB, lpxA, lpxB, lpxC, lpxD, lpxL, lpxM, ompA, pbpG* |
|  | Iron uptake systems | *barA, barB, basA, basB, basC, basD, basF, basG, basH, basI, basJ, bauA, bauB, bauC, bauD, bauE, bauF, entE* |
|  | Two-component systems | *bfmR, bfmS* |
| H2M2305 | Adherence | *fimT, fimU, fimV, gspO/pilD, pilA, pilB, pilC, pilE, pilF, pilG, pilH, pilI, pilJ, pilM, pilN, pilO, pilP, pilQ, pilR, pilS, pilT, pilU, pilV, pilW, pilX, pilY1, tsaP* |
|  | Quorum sensing and biofilm | *abaI, abaR, adeF, adeG, adeH, csuA, csuA/B, csuB, csuC, csuD, csuE, pgaA, pgaB, pgaC, pgaD* |
|  | Secretion system | *clpV/tssH, gspC, gspD, gspE1, gspE2, gspF, gspG, gspH, gspI, gspK, gspL, gspM, gspN, hcp/tssD, tagX, tssA, tssB, tssC, tssE, tssF, tssG, tssK, tssL, tssM, vgrG/tssI* |
|  | Exotoxin | *plc1, plc2, plcD* |
|  | Immune modulation | *galE, lpsB, lpxA, lpxB, lpxC, lpxD, lpxL, lpxM, ompA, pbpG* |
|  | Iron uptake systems | *barA, barB, basA, basB, basC, basD, basF, basG, basH, basI, basJ, bauA, bauB, bauC, bauD, bauE, bauF, entE* |
|  | Two-component systems | *bfmR, bfmS* |
| H2M2306 | Adherence | *fimT, fimU, fimV, gspO/pilD, pilA, pilB, pilC, pilE, pilF, pilG, pilH, pilI, pilJ, pilM, pilN, pilO, pilP, pilQ, pilR, pilS, pilT, pilU, pilV, pilW, pilX, pilY1, tsaP* |
|  | Quorum sensing and biofilm | *abaI, abaR, adeF, adeG, adeH, csuA, csuB, csuC, csuD, csuE, pgaA, pgaB, pgaC, pgaD* |
|  | Secretion system | *clpV/tssH, gspC, gspD, gspE1, gspE2, gspF, gspG, gspH, gspI, gspK, gspL, gspM, gspN, hcp/tssD, tagX, tssA, tssB, tssC, tssE, tssF, tssG, tssK, tssL, tssM, vgrG/tssI* |
|  | Exotoxin | *plc1, plc2, plcD* |
|  | Immune modulation | *galE, galU, lpsB, lpxA, lpxB, lpxC, lpxD, lpxL, lpxM, ompA, pbpG, pgi, pseB, pseC, pseF, pseG, pseH, pseI, tviB* |
|  | Iron uptake systems | *barA, barB, basA, basB, basC, basD, basF, basG, basH, basI, basJ, bauA, bauB, bauC, bauD, bauE, bauF, entE* |
|  | Two-component systems | *bfmR, bfmS* |
